# Factors associated with high-risk human papillomavirus infection and high-grade cervical neoplasia: A population-based study in Paraguay

**DOI:** 10.1371/journal.pone.0218016

**Published:** 2019-06-27

**Authors:** Elena Kasamatsu, María Isabel Rodríguez Riveros, Ana María Soilan, Marina Ortega, Pamela Mongelós, Malvina Páez, Amalia Castro, Carmen Cristaldo, Fátima Romina Báez, Claudia Carolina Centurión, Jaime Vester, Hernán Barrios, Griselda Villalba, María Luisa Amarilla, Graciela Giménez, Elodie Caubere, María de la Luz Hernández, Armando Baena, Maribel Almonte, Rolando Herrero, Laura Patricia Mendoza, for the ESTAMPA Paraguay Center study group

**Affiliations:** 1 Instituto de Investigaciones en Ciencias de la Salud, Universidad Nacional de Asunción (IICS-UNA), San Lorenzo, Paraguay; 2 Hospital Materno Infantil de San Lorenzo, Ministerio de Salud Pública y Bienestar Social, San Lorenzo, Paraguay; 3 Hospital Nacional, Ministerio de Salud Pública y Bienestar Social, Itauguá, Paraguay; 4 International Agency for Research on Cancer, IARC/WHO, Lyon, France; Greenebaum Cancer Center, Institute of Human Virology, University of Maryland School of Medicine, UNITED STATES

## Abstract

**Background:**

Cervical cancer (CC) is one of the leading causes of cancer mortality among women from Paraguay, with high incidence and mortality rates (31.2 and 16 per 100 000 women, respectively). Although the risk factors associated with high-risk human papillomavirus (hrHPV) infection and preneoplastic cervical lesions are widely studied, population-based characteristics of particular settings may influence the feasibility of HPV-based CC screening implementation. This study aimed to explore factors associated with hrHPV infection and high-grade cervical neoplasia in hrHPV-positive (hrHPV+) women from Paraguay.

**Methods:**

A total of 5677 women aged 30–64 years from the Central Department of Paraguay were screened with HPV test (Hybrid Capture 2) and Pap smear. Sociodemographic and risk factor interviews were conducted. hrHPV+ women were referred to colposcopy and women with an abnormal colposcopy had a biopsy taken. The outcomes recorded were the hrHPV status and the presence of high-grade cervical intraepithelial neoplasia or worse (CIN2+) among hrHPV+ women. Associations were investigated using multivariate logistic regressions.

**Results:**

hrHPV prevalence was 13.8% (95%CI 13.0–14.8). This value decreased with the age of women (p-trend<0.001) and increased with the lifetime number of sexual partners (p-trend<0.001) and number of previous female partners of their current male partner if women had had one lifetime sexual partner (p-trend<0.001), increasing from 3.06 (95%CI 0.073–20.9) if partners had had one previous female partner to 9.19 (95%CI 2.36–61.1) if they had had eight or more. In hrHPV+ women, CIN2+ prevalence was 10.7% (95%CI 8.58–13.2) and increased with time since the last Pap smear (p-trend<0.001) and with the increasing number of pregnancies (p-trend = 0.05).

**Conclusion:**

In these settings, the sexual behavior of women and their male partners is associated with hrHPV infection. In hrHPV+ women, underscreening practices and multiple pregnancies are associated with CIN2+. This knowledge can contribute to public health policies for CC prevention and control in Paraguay.

## Introduction

Cervical cancer (CC) ranks globally as the fourth most frequently diagnosed cancer and the fourth leading cause of cancer death in women, with an estimated 570 000 new cases and 311 000 deaths in 2018 [1] and more than 85% of the cases occurring in developing countries. In Paraguay, the burden of CC is a significant health problem with high incidence and mortality rates of 31.2 and 16 per 100 000 women, respectively [1].

It has been recognized that the main cause of CC and precancerous lesions is high-risk human papillomavirus (hrHPV) [2,3]. A worldwide study has shown that the most common HPV types associated with CC are HPVs 16, 18, 31 33, 35, 45, 52 and 58 [4], which account for 91% of the cases. In Paraguay, the most prevalent types in CC are HPVs 16, 18 and 45 (78.5%) [5,6], whereas the most prevalent ones in high-grade squamous intraepithelial lesions diagnosed by histology or cytology are HPVs 16, 18 and 33 [7]. HPV is one of the most common sexually transmitted infections. Most of the infections are transient and clear within two years without any treatment. Persistent infection with oncogenic hrHPV is considered as a necessary cause but not sufficient to develop cervical precancerous lesions and cancer [8,9]. Other cofactors such as multiparity, long-term hormonal contraceptive use, tobacco smoking and HIV infections, influence the risk of progression to high-grade squamous intraepithelial lesion or cancer in hrHPV infected women [10–12].

In low-and middle-income countries, cytology-based programs are difficult to implement and are characterized by low coverage and unsatisfactory follow-up. It has been demonstrated that HPV testing is substantially more sensitive than cytology for detection of high-grade cervical lesions and, although less specific, it has large potential to reduce CC rates [13–19]; thus, this method has been recommended by WHO as the primary cervical screening method to implement worldwide [20]. The national cytological screening program established in Paraguay more than 40 years ago has not been successful in controlling CC because of the low sensitivity of cytology, unsatisfactory follow-up of women with cytological abnormalities and the limited coverage reported in Paraguayan women (less than 20%) [21]. Currently the use of HPV testing is limited to private patients and to one of the reference public gynecology health centers but is not yet included in the national screening program for cervical cancer prevention and control.

This study is part of a large international project entitled “Multicentric study of cervical cancer screening and triage with human papillomavirus testing—ESTAMPA study” (NCT01881659) coordinated by the Prevention and Implementation Group of the International Agency for Research on Cancer/World Health Organization (IARC/WHO) and being conducted in nine countries of Latin America (12 centers), including Paraguay, since 2014. The aim of the ESTAMPA study is to investigate the performance of emerging CC screening and triage techniques in women aged 30–64 years old and to evaluate the feasibility of different approaches for the implementation of organized HPV-based screening programs. The target number of women to be recruited in the different research centers across Latin America is around 50 000.

In Paraguay, HPV types associated with CC and preneoplastic lesions have been previously studied but there are no population-based data or information on HPV cofactors. Therefore, within the framework of the ESTAMPA study, the aim of this study was to explore the prevalence and factors associated with hrHPV infection and CIN2+ in hrHPV+ women in the Central Department of Paraguay. The information collected may influence the feasibility of HPV-based cervical cancer screening implementation and could contribute to public health policies for CC prevention and control in Paraguay.

## Materials and methods

### Study design

This was a cross-sectional screening study, in which hrHPV testing was carried out in 5677 women aged 30–64 years old recruited in two districts of the Central Department of Paraguay during 2014–2018. All hrHPV+ women were referred to colposcopy, histological diagnosis and treatment as needed. The first outcome recorded was the hrHPV status of each woman, whereas the second one was the presence of high-grade cervical intraepithelial neoplasia or worse (CIN2+), including CIN2, CIN3 and CC among hrHPV+ women. To assure high-quality data and comparability between districts, standardized study forms and procedures, including monitoring and quality control were applied, based on the ESTAMPA protocol. Additionally, study investigators and coordinators received specific training in good clinical practice, field work, pathology, colposcopy, molecular biology and statistics.

The Central Department of Paraguay is made up of 19 districts and includes the city of Asunción, which is the capital of the country. According to data from the Dirección General de Estadística, Encuestas y Censos (DGEEC), in 2018 approximately 426 739 women aged 30 to 64 years were living in the Central Department. [22]

**Fig 1:**
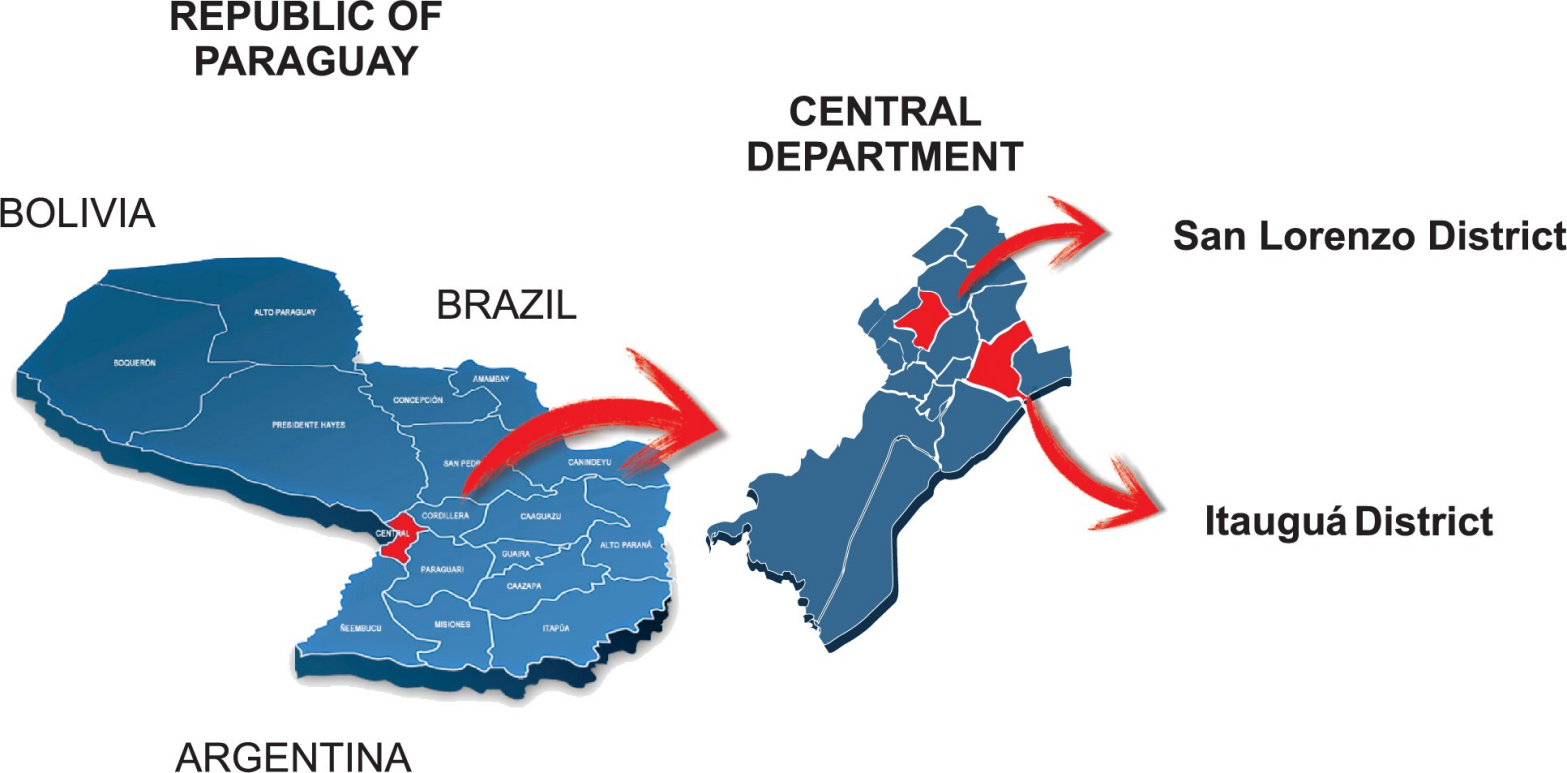
Map showing the political division of Paraguay, the location of Central Department and Itauguá and San Lorenzo Distrits.

The two districts mentioned above were selected for convenience from a well-defined catchment area of the Central Department with a baseline population of approximately 92 804 women aged 30 to 64 years. These districts included women of medium/low socioeconomic level and were close to primary health care centers as well as close to a referral hospital to facilitate the referral of hrHPV+ women to colposcopy visits and for treatment in case of CIN2+ (Fig 1). One of the selected districts was San Lorenzo, distant 14 km away from Asunción, the capital city of Paraguay where, with the collaboration of the Expanded Program on Immunization (EPI) the study group organized the census described below. In San Lorenzo, the referral Hospital Materno-Infantil de San Lorenzo, dependent on the Ministry of Public Health and Social Welfare of Paraguay (MSPyBS) collaborated with this study. The other district was Itauguá, 30km away from Asunción, including the urbanization developed by the National Housing Council of Paraguay (CONAVI) under the coverage of the referral Hospital Nacional de Itauguá, MSPyBS.

**Fig 2:**
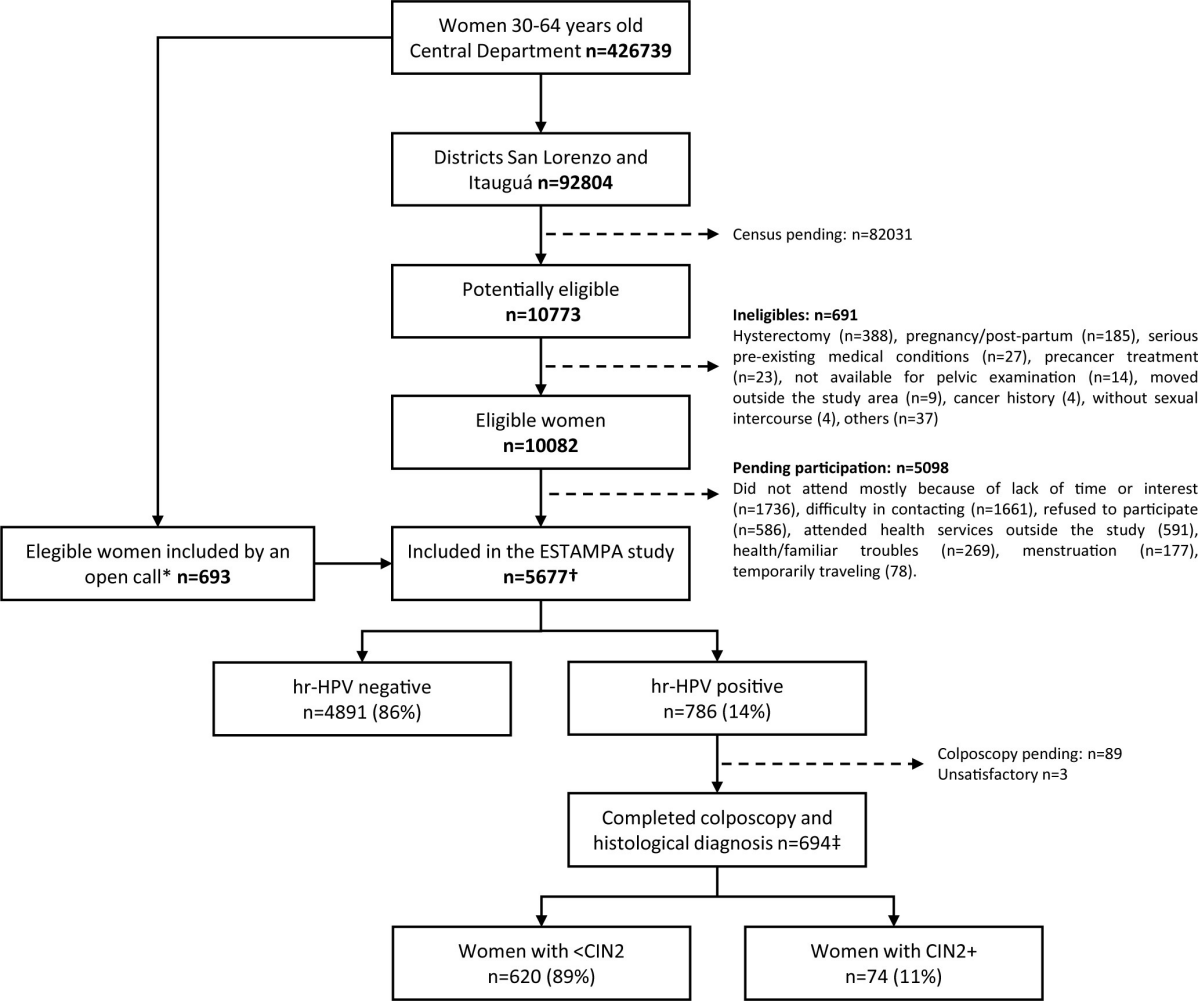
Flow diagram of participants. *Added participants enrolled by invitations (posters, talks) at the Universidad Nacional de Asunción, Paraguay. †Women included in the analysis for the first outcome: hrHPV status and associated risk factors. ‡Women included in the analysis for the second outcome: presence of CIN2+ and associated risk factors.

Information booklets were prepared and a door-to-door census was carried out, at which women or household members were informed about cervical screening, HPV testing, and the study. Potential eligible women aged 30 to 64 years old were invited to attend screening at the local clinic mainly by telephone calls. Women attending the clinic were then informed of the study and asked to sign an informed consent. A total of 691 out of 10 773 potentially eligible women were ineligible due to exclusion criteria such as hysterectomy, pregnancy/postpartum, not available for pelvic examination, pre-cancer treatment, no previous sexual intercourse, history of CC or serious pre-existing medical conditions (e.g. advanced cancer, terminal renal failure). Out of 10 082 potentially eligible women that fulfilled the inclusion criteria, 4984 accepted to participate in the study. Other 693 eligible women from the Central Department were also enrolled by invitation (poster, talks) at the Universidad Nacional de Asunción, Paraguay. Thus, a total of 5677 women were included in the study (Fig 2).

Eligible women were invited to participate in the study and scheduled to visit the nearest health center, through repeated phone calls. At the health center, after initial information and orientation about the study and the signature of the informed consent, trained staff administered a socio-demographic and risk factor questionnaires (Supporting information S1–S6 Files) to obtain data on years of education, year of last Pap smear, age at first sexual intercourse, lifetime number of sexual partners, number of previous female partners of their current male partner, number of pregnancies, use of contraceptive methods, cigarette use and years and lifetime number of cigarettes smoked. Then, trained nurses performed a pelvic exam and cervical sample collection for Pap smear and detection of hrHPV. The samples collected were labeled and sent to the laboratory of the Instituto de Investigaciones en Ciencias de la Salud, Universidad Nacional de Asunción (IICS-UNA) for processing. hrHPV+ women were adequately informed and invited to have a colposcopy done. hrHPV-negative (hrHPV-) women returned to regular screening with Pap smear, offered free of charge by the MSPyBS.

### hrHPV detection

Cervical samples for hrHPV testing were collected in PreservCyt medium and 4 ml of each residual PreservCyt sample was centrifuged and resuspended in 150 μl of a mixture of specimen transport medium and denaturation buffer provided in the kit. A 75 μl aliquot of each resuspended sample was used for each Hybrid Capture 2 assay, a sandwich capture molecular hybridization assay that uses chemiluminescence detection. All assays were performed according to the manufacturer’s protocol, with the results reported as a ratio of relative light units (RLU/CO). Samples with an RLU/CO ratio >1.0 were considered positive for hrHPV.

### Colposcopy, histopathological study and clinical management

All hrHPV+ women were referred to colposcopy for diagnosis and treatment by two colposcopists trained in the study protocol. In women with abnormal colposcopic findings, 2–3 biopsies from the aceto-white lesions observed were taken, fixed in 10% neutral buffered formalin and transported to a pathology laboratory for histological procedures. Histologically, cervical lesions were classified as normal, CIN1, CIN2, CIN3, and CC. The final histopathological diagnosis was based on the highest grade of the lesion observed in biopsy or in the histological specimen of the large loop excision of the transformation zone (LLETZ) of each woman. All histology samples were read by the local pathologist experienced in CC pathology trained in the study protocol. Women with CIN2+ were treated with LLETZ and those with CC were referred to a tertiary care center. Women with negative colposcopy or histology less than CIN2 (<CIN2) were invited to a follow-up hrHPV screening at 18 months, whereas those with positive colposcopy or CIN2+ had a final colposcopy. Women with histological CIN2+ were treated adequately and hrHPV- women or with <CIN2 are given clear follow-up indications to continue with regular screening within the local health care system.

### Statistical analysis

The main outcomes were the hrHPV infection status of each woman and the CIN2+ lesions present in hrHPV+ women. Bivariate analyses between sociodemographic characteristics and known risk factors (independent variables) for hrHPV infection and CIN2+ were conducted using Chi-square or Mann-Whitney tests for categorical or numerical variables, respectively. Age-adjusted and multivariate-adjusted odds ratios (OR) with their corresponding 95% confidence intervals (CI) were estimated from logistic regressions to explore associations between independent variables and outcomes. Independent variables regarding well-established risk factors for both hrHPV infection (sexual behavior variables) [23,24] and CIN2+ (number of pregnancies, cigarette consumption and contraceptive use) [25,26] were selected for the corresponding multivariate analyses adjusted by age, education level and screening history (time since the last cytology). Herein we included age of first intercourse, use of condom and lifetime number of sexual partners for the hrHPV multivariate analysis, and number of pregnancies, years of cigarette use and years of hormonal contraceptive use for the CIN2+ multivariate analysis. Additionally, we evaluated whether the sexual history of the current partner of women with only one lifetime sexual partner was associated with hrHPV infection. Therefore, an additional analysis among women with only one lifetime sexual partner was conducted. Analyses were performed in R (R Core Team (2018). R: A language and environment for statistical computing. R Foundation for Statistical Computing, Vienna, Austria. URL https://www.R-project.org/) and all tests were two-sided with a significance level of 0.05.

### Ethical considerations

The protocol was approved by the Ethics Committee of the International Agency for Research on Cancer, World Health Organization (IARC/WHO), Pan American Health Organization (PAHO) and by IICS/UNA.

## Results

### Characteristics of the study population

A total of 5677 of the 10 082 30-64-year-old eligible women and 693 women invited by the UNA were enrolled. Among them, 4354 women were from the San Lorenzo district and 630 women from the Itauguá district. The distribution of socio-demographic, reproductive and sexual behavioral characteristics of the study population is presented in Table 1. The mean/SD age was 44.5/9.25. A total of 5496 (96.9%) reported having had Pap smears and almost half of these women (48.1%) had the last Pap smear in the previous 2 years. However, 1375 (24.2%) had the last Pap smear >5 years or never before the interview; 4246 (74.8%) had high school education or less, 2978 (52.5%) had their first intercourse at 17–20 years, 3925 (69.3%) had ≥2 lifetime sexual partners 438 (25.2%) out of the 1739 women with one lifetime sexual partner answered about the number of previous female partners of their current male partner, 2428 (42.8%) had 2–3 pregnancies and 4844 (85.3%) never smoked. Regarding contraceptive methods, 3145 (55.5%) never used condoms and 3850 (67.8%) used hormonal contraceptives, among which 2643 (46.6%) reported using them for less than 10 years.

**Table 1:** Characteristics and prevalence of hrHPV in 30-69-year-old women from the Central Department Paraguay.

Characteristic		Overall		hrHPV-	hrHPV+	Prevalence (%) of		P*
		N	(%)	N	N	hrHPV (95% CI)		
Total		5677	-100	4891	786	13.8	(13.0–14.8)	
**Age, yr**								
	*[Mean/SD]*	*[44*.*5/9*.*25]*		*[44*.*8/9*.*19]*	*[42*.*7/9*.*48]*			*<0*.*001*
	30–34	852	15	672	180	21.1	(18.5–24.0)	<0.001
	35–39	1116	19.7	950	166	14.9	(12.9–17.1)	
	40–44	915	16.1	803	112	12.2	(10.3–14.5)	
	45–49	947	16.7	830	117	12.4	(10.4–14.6)	
	50–54	780	13.7	700	80	10.3	(8.32–12.6)	
	55–59	609	10.7	537	72	11.8	(9.49–14.6)	
	60–64	458	8.07	399	59	12.9	(10.1–16.3)	
	Missing data	0		0	0			
**Have ever had a pap smear**								
	Yes	5496	96.9	4744	752	13.7	(12.8–14.6)	0.07
	No	175	3.09	142	33	18.9	(13.8–25.3)	
	Missing data	6		5	1			
**Last pap smear, yr**								
	<2	2730	48.1	2367	363	13.3	(12.1–14.6)	0.24
	2–5	1566	27.6	1353	213	13.6	(12.0–15.4)	
	>5 or never	1375	24.2	1166	209	15.2	(13.4–17.2)	
	Missing data	6		5	1			
**Education**								
	Incomplete elementary school	1057	18.6	908	149	14.1	(12.1–16.3)	0.99
	Complete elementary school	1141	20.1	981	160	14	(12.1–16.2)	
	Incomplete high school	902	15.9	778	124	13.7	(11.7–16.1)	
	Complete high school	1146	20.2	993	153	13.4	(11.5–15.4)	
	Higher education	1431	25.2	1,231	200	14	(12.3–15.9)	
	Missing data	0		0	0			
**Age of first intercourse**								
	*[Mean/SD]*	*[18*.*6/3*.*41]*		*[18*.*6/3*.*41]*	*[18*.*4/3*.*42]*			
	≥21	1201	21.2	1043	158	13.2	(11.4–15.2)	0.1
	17–20	2978	52.5	2582	396	13.3	(12.1–14.6)	
	≤16	1497	26.4	1265	232	15.5	(13.8–17.4)	
	Missing data	1		1	0			
**Use of condom**								
	Yes	2523	44.5	2184	339	13.4	(12.2–14.8)	0.42
	No	3145	55.5	2698	447	14.2	(13.0–15.5)	
	Missing data	9		9	0			
**Frequency of condom use**								
	Always	495	8.74	427	68	13.7	(11.0–17.1)	0.6
	Most of the times	346	6.11	296	50	14.5	(11.1–18.5)	
	Sometimes	968	17.1	850	118	12.2	(10.3–14.4)	
	Rarely	710	12.5	609	101	14.2	(11.8–17.0)	
	Never	3145	55.5	2698	447	14.2	(13.0–15.5)	
	Missing data	13		11	2			
**Lifetime number of sexual partners**								
	*[Median/IQR]*	*[2*.*00/3*.*00]*		*[2*.*00/2*.*00]*	*[3*.*00/2*.*00]*			*<0*.*001*
	1	1739	30.7	1548	191	11	(9.60–12.5)	<0.001
	2–3	2482	43.8	2113	369	14.9	(13.5–16.3)	
	4–5	936	16.5	812	124	13.2	(11.2–15.6)	
	≥6	507	8.95	406	101	19.9	(16.7–23.6)	
	Missing data	13		12	1			
**Different sexual partners during the last year**								
	*[Median/IQR]*	*[1*.*00/1*.*00]*		*[1*.*00/1*.*00]*	*[1*.*00/1*.*00]*			*<0*.*001*
	0	2049	36.4	1815	234	11.4	(10.1–12.9)	<0.001
	1	3443	61.1	2934	509	14.8	(13.6–16.0)	
	≥2	140	2.49	104	36	25.7	(19.2–33.5)	
	Missing data	45		38	7			
**Number of previous female partners of current partner**								
	*[Median/IQR]*	*[2*.*50/3*.*50]*		*[2*.*50/3*.*50]*	*[3*.*00/4*.*50]*			*0*.*002*
	0	63	1.11	61	2	3.17	(0.87–10.9)	<0.001
	1	95	1.68	87	8	8.42	(4.33–15.7)	
	2–3	141	2.49	120	21	14.9	(9.95–21.7)	
	4–7	78	1.38	64	14	17.9	(11.0–27.9)	
	≥8	61	1.08	48	13	21.3	(12.9–33.1)	
	Does not know / did not answer	1301	23	1168	133	10.2	(8.69–12.0)	
	Not asked**	3924	69.3	3330	594	15.1	(14.1–16.3)	
	Missing data	14		13	1			
**Sexual intercourse during the last year**								
	No	239	4.44	212	27	11.3	(7.88–15.9)	0.28
	Yes	5149	95.6	4428	721	14	(13.1–15.0)	
	Missing data	289		251	38			
**Ever pregnant**								
	No	241	4.25	197	44	18.3	(13.9–23.6)	0.05
	Yes	5433	95.8	4691	742	13.7	(12.8–14.6)	
	Missing data	3		3	0			
**Number of pregnancies**								
	*[Median/IQR]*	*[3*.*00/2*.*00]*		*[3*.*00/2*.*00]*	*[3*.*00/2*.*75]*			*0*.*86*
	0–1	977	17.2	832	145	14.8	(12.7–17.2)	0.36
	2–3	2428	42.8	2109	319	13.1	(11.9–14.5)	
	≥4	2269	40	1947	322	14.2	(12.8–15.7)	
	Missing data	3		3	0			
**Cigarette use**								
	Never	4844	85.3	4173	671	13.9	(12.9–14.9)	1
	Ever	833	14.7	718	115	13.8	(11.6–16.3)	
	Missing data	0		0	0			
**Years of cigarette use**								
	*[Mean/SD]*	*[1*.*94/6*.*57]*		*[1*.*97/6*.*66]*	*[1*.*73/6*.*01]*			*0*.*74*
	0	4930	87	4245	685	13.9	(13.0–14.9)	0.59
	<10	291	5.13	247	44	15.1	(11.5–19.7)	
	≥10	447	7.89	391	56	12.5	(9.77–15.9)	
	Missing data	9		8	1			
**Lifetime number of cigarettes**								
	0	4930	87.2	4245	685	13.9	(13.0–14.9)	0.93
	<15k	407	7.2	353	54	13.3	(10.3–16.9)	
	≥15k	317	5.61	274	43	13.6	(10.2–17.8)	
	Missing data	23		19	4			
**Use of hormonal contraceptives**								
	No	1826	32.2	1595	231	12.7	(11.2–14.3)	0.08
	Yes	3850	67.8	3295	555	14.4	(13.3–15.6)	
	Missing data	1		1	0			
**Years of hormonal contraceptive use**								
	*[Median/IQR]*	*[2*.*00/8*.*00]*		*[2*.*00/8*.*00]*	*[2*.*00/8*.*00]*			*0*.*3*
	0	1826	32.2	1595	231	12.7	(11.2–14.3)	0.18
	<10	2643	46.6	2258	385	14.6	(13.3–16.0)	
	≥10	1207	21.3	1037	170	14.1	(12.2–16.2)	
	Missing data	1		1	0			

%: column percentages for the characteristics of the overall population.

*Pearson's chi-squared test or Mann-Whitney test to compare categorical or numerical variables, respectively, between hrHPV-positive and -negative women.

**Not asked to women responding to having had more than one lifetime sexual partner.

CI: Confidence Interval. SD: Standard Deviation. IQR: Interquartile Range.

### Prevalence of hrHPV infection and CIN2+ in hrHPV+ women

The overall hrHPV prevalence was 13.8% (95%CI 13.0–14.8). The highest prevalence of hrHPV infection was observed among women aged 30–34 years (21.12%, 95%CI 18.5–24.0) and decreased with increasing age to 11.8% (95%CI 9.49–14.6) in 55-59-year-old women (Table 1).

Among the 694 hrHPV+ women with histopathological diagnosis, 74 (10.7%, 95%CI 8.58–13.2) had CIN2+. The mean/SD age of CIN2+ women was 40.2/7.42, with higher prevalences in women aged 35–39 years (14.1%, 95%CI 9.31–20.8), 40–44 years (13.6%, 95%CI 8.27–21.5) and 50–54 years (13.7%, 95%CI 7.61–23.4) (Table 2). The histopathological diagnosis of CIN2+ showed 8 cases of CIN2, 55 CIN 3 and 11 CC.

**Table 2:** Characteristics and prevalence of CIN2+ in 30-69-year-old hrHPV+ women with histopathological diagnosis from the Central Department, Paraguay.

Characteristic		Overall		<CIN2	CIN2+	Prevalence (%) of		P*
		N	(%)	n	N	CIN2+ (95% CI)		
Total		694	100	620	74	10.7	(8.58–13.2)	
**Age, yr**								
	*[Mean/SD]*	*[42*.*7/9*.*44]*		*[43*.*0/9*.*64]*	*[40*.*9/7*.*42]*			*0*.*19*
	30–34	158	22.8	143	15	9.49	(5.84–15.1)	0.09
	35–39	142	20.5	122	20	14.1	(9.31–20.8)	
	40–44	103	14.8	89	14	13.6	(8.27–21.5)	
	45–49	103	14.8	92	11	10.7	(6.07–18.1)	
	50–54	73	10.5	63	10	13.7	(7.61–23.4)	
	55–59	67	9.65	63	4	5.97	(2.35–14.4)	
	60–64	48	6.92	48	0	0	(0.00–7.41)	
	Missing data	0		0	0			
**Have ever had a pap smear**								
	Yes	663	95.5	592	71	10.7	(8.58–13.3)	1
	No	31	4.47	28	3	9.68	(3.35–24.9)	
	Missing data	0		0	0			
**Last pap smear, yr**								
	<2	331	47.7	305	26	7.85	(5.42–11.3)	0.002
	2–5	182	26.2	166	16	8.79	(5.48–13.8)	
	>5 or never	181	26.1	149	32	17.7	(12.8–23.9)	
	Missing data	0		0	0			
**Education**								
	Incomplete elementary school	130	18.7	114	16	12.3	(7.72–19.1)	0.55
	Complete elementary school	141	20.3	123	18	12.8	(8.23–19.3)	
	Incomplete high school	113	16.3	104	9	7.96	(4.25–14.4)	
	Complete high school	133	19.2	117	16	12	(7.54–18.6)	
	Higher education	177	25.5	162	15	8.47	(5.20–13.5)	
	Missing data	0		0	0			
**Age of first intercourse**								
	*[Mean/SD]*	*[18*.*5/3*.*52]*		*[18*.*5/3*.*61]*	*[17*.*8/2*.*47]*			
	≥21	141	20.3	132	9	6.38	(3.39–11.7)	0.16
	17–20	349	50.3	306	43	12.3	(9.28–16.2)	
	≤16	204	29.4	182	22	10.8	(7.23–15.8)	
	Missing data	0		0	0			
**Use of condom**								
	Yes	305	43.9	272	33	10.8	(7.81–14.8)	1
	No	389	56.1	348	41	10.5	(7.86–14.0)	
	Missing data	0		0	0			
**Frequency of condom use**								
	Always	58	8.38	53	5	8.62	(3.74–18.6)	0.37
	Most of the time	43	6.21	40	3	6.98	(2.40–18.6)	
	Sometimes	108	15.6	91	17	15.7	(10.1–23.8)	
	Rarely	94	13.6	86	8	8.51	(4.38–15.9)	
	Never	389	56.2	348	41	10.5	(7.86–14.0)	
	Missing data	2		2	0			
**Lifetime number of sexual partners**								
	*[Median/IQR]*	*[3*.*00/3*.*00]*		*[3*.*00/3*.*00]*	*[3*.*00/2*.*00]*			*0*.*23*
	1	174	25.1	158	16	9.2	(5.74–14.4)	0.64
	2–3	319	46	287	32	10	(7.20–13.8)	
	4–5	108	15.6	94	14	13	(7.88–20.6)	
	≥6	92	13.3	80	12	13	(7.62–21.4)	
	Missing data	1		1	0			
**Different sexual partners during the last year**								
	*[Median/IQR]*	*[1*.*00/1*.*00]*		*[1*.*00/1*.*00]*	*[1*.*00/1*.*00]*			*0*.*86*
	0	206	29.9	184	22	10.7	(7.16–15.6)	0.75
	1	452	65.7	402	50	11.1	(8.49–14.3)	
	≥2	30	4.36	28	2	6.67	(1.85–21.3)	
	Missing data	6		6	0			
**Number of previous female partners of current partner**								
	*[Median/IQR]*	*[3*.*00/4*.*50]*		*[2*.*75/4*.*38]*	*[6*.*50/7*.*75]*			*0*.*19*
	0	2	0.29	2	0	0	(0.00–65.8)	0.92
	1	7	1.01	6	1	14.3	(0.73–51.3)	
	2–3	19	2.74	18	1	5.26	(0.27–24.6)	
	4–7	14	2.02	13	1	7.14	(0.37–31.5)	
	≥8	12	1.73	10	2	16.7	(4.70–44.8)	
	Does not know / did not answer	120	17.3	109	11	9.17	(5.20–15.7)	
	Not asked**	519	74.9	461	58	11.2	(8.74–14.2)	
	Missing data	1		1	0			
**Sexual intercourse during the last year**								
	No	24	3.61	24	0	0	(0.00–13.8)	0.18
	Yes	641	96.4	573	68	10.6	(8.45–13.2)	
	Missing data	29		23	6			
**Ever pregnant**								
	No	39	5.62	37	2	5.13	(1.42–16.9)	0.38
	Yes	655	94.4	583	72	11	(8.82–13.6)	
	Missing data	0		0	0			
**Number of pregnancies**								
	*[Median/IQR]*	*[3*.*00/2*.*00]*		*[3*.*00/2*.*00]*	*[3*.*00/3*.*00]*			*0*.*03*
	0–1	128	18.4	121	7	5.47	(2.67–10.9)	0.09
	2–3	282	40.6	251	31	11	(7.85–15.2)	
	≥4	284	40.9	248	36	12.7	(9.30–17.0)	
	Missing data	0		0	0			
**Cigarette use**								
	Never	590	85	531	59	10	(7.83–12.7)	0.24
	Ever	104	15	89	15	14.4	(8.94–22.4)	
	Missing data	0		0	0			
**Years of cigarette use**								
	*[Mean/SD]*	*[1*.*77/6*.*04]*		*[1*.*70/6*.*05]*	*[2*.*33/5*.*97]*			*0*.*11*
	0	603	87	543	60	9.95	(7.81–12.6)	0.21
	<10	39	5.63	34	5	12.8	(5.60–26.7)	
	≥10	51	7.36	42	9	17.6	(9.57–30.3)	
	Missing data	1		1	0			
**Lifetime number of cigarettes**								
	0	603	87.4	543	60	9.95	(7.81–12.6)	0.37
	<15k	47	6.81	40	7	14.9	(7.41–27.7)	
	≥15k	40	5.8	34	6	15	(7.06–29.1)	
	Missing data	4		3	1			
**Use of hormonal contraceptives**								
	No	206	29.7	191	15	7.28	(4.46–11.7)	0.08
	Yes	488	70.3	429	59	12.1	(9.49–15.3)	
	Missing data	0		0	0			
**Years of hormonal contraceptive use**								
	*[Median/IQR]*	*[2*.*00/8*.*00]*		*[2*.*00/8*.*00]*	*[4*.*83/9*.*19]*			*0*.*07*
	0	206	29.7	191	15	7.28	(4.46–11.7)	0.17
	<10	335	48.3	295	40	11.9	(8.89–15.9)	
	≥10	153	22	134	19	12.4	(8.10–18.6)	
	Missing data	0		0	0			

%: column percentages for the characteristics of the overall population.

*Pearson's chi-squared test or Mann-Whitney test to compare categorical or numerical variables, respectively, between CIN2+ and <CIN2 women.

**Not asked to women responding to having had more than one lifetime sexual partner.

CI: Confidence Interval. SD: Standard Deviation. IQR: Interquartile Range.

### Risk factors for hrHPV infection and CIN2+ among hrHPV+ women

Risk factors for hrHPV infection were investigated in 5677 women, whereas risk factors for CIN2+ were analyzed in 694 hrHPV+ women (Fig 2). Table 3 shows the risk factors associated with hrHPV infection. The multivariable adjusted analysis showed that the risk of hrHPV infection decreased with the age of the woman (p-trend<0.001) and increased with the number of lifetime sexual partners, compared ≥6 partners with one partner (OR = 1.81, 95%CI 1.37–2.38, p-trend<0.001). Among the 1739 women with one lifetime partner the risk of hrHPV infection increased with the number of previous female partners of their current male partner (p-trend<0.001).

**Table 3:** Odds ratios of hrHPV associated with sociodemographic characteristics and selected risk factors among 30-69-year-old women from the Central Department, Paraguay.

Characteristic		OR†	(95% CI)	P*	OR‡	(95% CI)	P*
**Age, yr**							
	30–34	1			1		
	35–39	0.65	(0.52–0.82)	<0.001	0.65	(0.52–0.83)	<0.001
	40–44	0.52	(0.40–0.67)		0.52	(0.40–0.67)	
	45–49	0.53	(0.41–0.68)		0.51	(0.39–0.66)	
	50–54	0.43	(0.32–0.57)		0.41	(0.30–0.54)	
	55–59	0.50	(0.37–0.67)		0.48	(0.35–0.65)	
	60–64	0.55	(0.40–0.76)		0.52	(0.37–0.73)	
	*P-trend*	*<0*.*001*			*<0*.*001*		
**Last pap smear, yr**							
	<2	1			1		
	2–5	1.01	(0.84–1.22)	0.24	1.00	(0.84–1.21)	0.05
	>5 or never	1.24	(1.03–1.50)		1.22	(1.01–1.47)	
	*P-trend*	*0*.*04*			*0*.*06*		
**Education**							
	Incomplete elementary school	1			1		
	Complete elementary school	0.96	(0.75–1.23)	0.99	1.00	(0.78–1.27)	0.53
	Incomplete high school	0.88	(0.68–1.14)		0.88	(0.67–1.15)	
	Complete high school	0.81	(0.63–1.04)		0.84	(0.65–1.09)	
	Higher education	0.83	(0.65–1.06)		0.87	(0.68–1.13)	
	*P-trend*	*0*.*06*			*0*.*15*		
**Age of first intercourse**							
	≥21	1			1		
	17–20	0.97	(0.79–1.18)	0.10	0.89	(0.72–1.09)	0.41
	≤16	1.12	(0.90–1.40)		0.96	(0.76–1.23)	
	*P-trend*	*0*.*24*			*0*.*91*		
**Use of condom**							
	Yes	1			1		
	No	1.16	(0.99–1.36)	0.40	1.11	(0.95–1.30)	0.14
**Lifetime number of sexual partners**							
	1	1			1		
	2–3	1.37	(1.14–1.66)	<0.001	1.38	(1.14–1.67)	<0.001
	4–5	1.15	(0.90–1.47)		1.17	(0.91–1.49)	
	≥6	1.81	(1.38–2.37)		1.83	(1.39–2.41)	
	*P-trend*	*<0*.*001*			*<0*.*001*		
**Number of previous female partners of current partner**							
	0	1					
	1	2.94	(0.60–14.4)	0.004			
	2–3	5.66	(1.28–25.0)				
	4–7	7.84	(1.70–36.2)				
	≥8	9.15	(1.95–42.8)				
	Does not know / Did not answer	3.70	(0.89–15.4)				
	Not asked**	—					
	*P-trend*	*<0*.*001§*					

†Odds ratio (OR) adjusted by age.

‡OR adjusted by age, last pap smear, education, age of first intercourse, use of condom and lifetime number of sexual partners.

*Likelihood-ratio chi-squared test.

**Not asked to women responding to having had more than one lifetime sexual partner.

CI: confidence interval.

§Excludes 'Does not know' category.

Comparison of women with one lifetime sexual partner whose partners had not had previous female partners showed that the adjusted OR of having hrHPV increased from 3.06 (95%CI 0.73–20.9) if partners had had one previous female partner to 9.19 (95%CI 2.36–61.1) if they had had ≥8 (Table 4).

**Table 4:** Odds ratios of hrHPV infection associated with sociodemographic characteristics and selected risk factors among 30-69-year-old women with one lifetime sexual partner from the Central Department, Paraguay.

Characteristic		OR‡	(95% CI)	P*
**Age, yr**				
	30–34	1		
	35–39	0.6	(0.34–1.04)	0.02
	40–44	0.46	(0.25–0.82)	
	45–49	0.41	(0.23–0.74)	
	50–54	0.42	(0.23–0.75)	
	55–59	0.46	(0.25–0.84)	
	60–64	0.85	(0.47–1.53)	
	*P-trend*	*0*.*39*		
**Last pap smear, yr**				
	<2	1		
	2–5	0.87	(0.58–1.28)	0.24
	>5 or never	1.19	(0.83–1.71)	
	*P-trend*	*0*.*43*		
**Education**				
	Incomplete primary	1		
	Complete primary	0.87	(0.54–1.38)	0.88
	Incomplete secondary	0.86	(0.50–1.45)	
	Complete secondary	0.84	(0.51–1.37)	
	Higher education	0.79	(0.48–1.31)	
	*P-trend*	*0*.*39*		
**Age of first intercourse**				
	≥21	1		
	17–20	1.08	(0.75–1.55)	0.8
	≤16	0.93	(0.57–1.50)	
	*P-trend*	*0*.*86*		
**Use of condom**				
	Yes	1		
	No	1.1	(0.79–1.53)	0.52
**Number of previous female partners of current partner**				
	0	1		
	1	3.06	(0.73–20.9)	0.002
	2–3	5.78	(1.60–37.1)	
	4–7	8.07	(2.11–53.1)	
	≥8	9.19	(2.36–61.1)	
	Does not know / did not answer	3.78	(1.15–23.3)	
	Not asked**	—		
	*P-trend*	*<0*.*001§*		

‡Odds ratio (OR) adjusted by age, last pap smear, education, age of first intercourse, use of condom and additional female partners of regular partner; multivariate model is restricted to n = 1,739 women with one lifetime sexual partner. Likelihood-ratio chi-squared test.

*Likelihood-ratio chi-squared test.

**Not asked to women responding to having had more than one lifetime sexual partner.

CI: confidence interval.

§Excludes 'Does not know' category.

The time of the last Pap smear, education level, age of first intercourse, having had different sexual partners during the last year, number of pregnancies, cigarette use, condom use and hormonal contraceptive use were not significantly associated with hrHPV infection.

Among hrHPV+ women, the risk of CIN2+ increased with the time of the last Pap smear (p-trend<0.001). As compared with women who had had a Pap smear in the last two years, those having had a Pap smear within 2–5 years were at increased risk (OR = 1.25, 95%CI 0.62–2.44), with a further increase in risk of CIN2+ for women with more than 5 years since the last Pap smear (OR = 3.00, 95%CI 1.67–5.47). In addition, the risk of CIN2+ was higher among women with ≥4 pregnancies (OR = 2.72, 95%CI 1.11–7.45) than among those with 0–1 pregnancy (p-trend = 0.05). The age, education level, years of hormonal contraceptive use and years of cigarette use were not significantly associated with the risk of CIN2+ (Table 5).

**Table 5:** Odds ratios of CIN2+ associated with sociodemographic characteristics and selected risk factors among 30-69-year-old hrHPV+ women from the Central Department, Paraguay.

Characteristic		OR†	(95% CI)	P*	OR‡	(95% CI)	P*
**Age, yr**							
	30–34		1			1	
	35–39	1.56	(0.77–3.18)	0.01	1.41	(0.67–3.05)	0.03
	40–44	1.50	(0.69–3.25)		1.01	(0.43–2.36)	
	45–49	1.14	(0.50–2.59)		0.86	(0.34–2.10)	
	50–54	1.51	(0.64–3.55)		0.96	(0.37–2.44)	
	55–59	0.61	(0.19–1.90)		0.40	(0.10–1.25)	
	60–64	0.00	—		0.00	—	
	*P-trend*	*0*.*09*			*0*.*008*		
**Last pap smear, yr**							
	<2	1			1		
	2–5	1.13	(0.59–2.19)	0.003	1.25	(0.62–2.44)	<0.001
	>5 or never	2.76	(1.57–4.85)		3.00	(1.67–5.47)	
	*P-trend*	*<0*.*001*			*<0*.*001*		
**Education**							
	Incomplete elementary school	1			1		
	Complete elementary school	0.83	(0.39–1.74)	0.54	0.71	(0.32–1.55)	0.39
	Incomplete high school	0.48	(0.20–1.17)		0.49	(0.19–1.21)	
	Complete high school	0.71	(0.33–1.54)		0.70	(0.31–1.61)	
	Higher education	0.44	(0.20–0.97)		0.58	(0.25–1.36)	
	*P-trend*	*0*.*05*			*0*.*28*		
**Number of pregnancies**							
	0–1	1			1		
	2–3	2.16	(0.92–5.07)	0.06	2.12	(0.92–5.58)	0.04
	≥4	3.04	(1.26–7.32)		2.72	(1.11–7.45)	
	*P-trend*	*0*.*01*			*0*.*05*		
**Years of cigarette use**							
	0	1			1		
	<10	1.45	(0.54–3.91)	0.25	1.69	(0.53–4.48)	0.25
	≥10	2.15	(0.98–4.73)		1.85	(0.77–4.07)	
	*P-trend*	*0*.*05*			*0*.*10*		
**Years of hormonal contraceptive use**							
	0	1			1		
	<10	1.57	(0.84–2.96)	0.15	1.40	(0.73–2.78)	0.48
	≥10	1.61	(0.78–3.31)		1.53	(0.72–3.30)	
	*P-trend*	*0*.*19*			*0*.*27*		

†Odds ratio (OR) adjusted by age.

‡OR adjusted by age, last pap smear, education, number of pregnancies, years of cigarette use and years of hormonal contraceptive use.

*Likelihood-ratio chi-squared test.

CI: Confidence Interval. —: Did not converge due to insufficient number of observations.

## Discussion

This is the first population-based study of hrHPV infection and CC precursors using hrHPV testing in Paraguay, which assesses the prevalence and risk factors of hrHPV infection and CIN2+ in hrHPV+ women living in two districts of the Central Department. The results showed that the prevalence of hrHPV infection (13.8%) was consistent with that observed in Paraguayan women without cytological abnormalities in five indigenous communities of Paraguayan Chaco (16.1%) and those coming from reference health centers in the Central Department, of Paraguay (13.5%). [7,27]. The higher hrHPV prevalence observed in indigenous women could be due to the fact that the median age was lower (30) than that of women enrolled in the present study (44.5).

The prevalence of HPV differs worldwide, with higher prevalence values in Sub-Saharan African regions (24%), Latin America and the Caribbean (16.1%), Eastern Europe (14.2%) and Southeastern Asia (14%) [28]. The hrHPV prevalence found in the present study in Paraguayan women is comparable to that found in more recent results from Latin America and the Caribbean centers participating in the ESTAMPA study, using comparable methodologies (Almonte, personal communication).

This study showed decreasing hrHPV prevalence with increasing age, consistent with previous studies [28–31]. A non-significant small second peak was observed in postmenopausal women aged 60–64. This second peak at perimenopausal or postmenopausal ages has been previously described in Latin American women and could be related to a cohort effect due to higher HPV exposure of older women when they were young, to the reactivation of latent HPV infection by decreased immune response or hormonal changes or to new HPV infection with an increase of low risk HPV [29, 32–35].

In the present study, we also explored the risk factors for hrHPV infection and found a strong association with the lifetime number of sexual partners. In Latin America and the Caribbean, the acquisition of HPV infection is associated with an increased number of lifetime sexual partners as well as with the number of recent sexual partners [5,16,29,33,36]. A pooled analysis of the IARC HPV surveys of 11 337 women from Vietnam, Thailand, Korea, Nigeria, Spain, Argentina, Chile, Mexico and Colombia, confirmed that the lifetime number of sexual partners was the most important risk factor for HPV infection and that the heterogeneity in the ORs for HPV positivity associated with multiple sexual partners is probably due to the fact that some women underreport their lifetime number of sexual partners, which is more severe in the traditional Asian Society than in Europe, Africa or Latin America [23]. In addition, a clear increase in risk was observed with the number of previous female partners of the current male partners of women with one lifetime sexual partner. The strongest association was observed for partners with eight or more additional female partners (OR = 9.19, 95%CI 2.36–61.1; p-trend<0.001) compared to women who reported that their partners had had only one lifetime sexual partner. From our point of view, considering the cultural characteristics of Paraguayan women, including their language (guaraní language), well trained study field workers made all efforts and collect as adequate as possible information about the number of lifetime sexual partners, different sexual partners during the last year and number of previous female partners of their husband or current male partner in women with one lifetime sexual partner. However, we can not exclude potential reporting bias, even if the woman assures to know the sexual behavior of her partner.

In hrHPV+ women, we observed a strong association between CIN2+ and the time to their last Pap smear, particularly in those who had had their last Pap smear more than 5 years before the interview (OR = 3.00, 95% CI 1.67–5.47; p-trend<0.001). A previous study on CC in Paraguay, showed that never having had a Pap smear was one of the main risk factors for CC (OR = 26.7, 95%CI 2.4–296.9) [5]. This finding is important for CC prevention and control program in Paraguay, in which the cytology-based national screening program is opportunistic, with limited coverage (20%) [21], although Pap testing has been free of charge for many years. Also, this knowledge can contribute to supporting the incorporation of hrHPV testing, that allows extension of screening intervals as the primary screening method in our country.

Our results also showed that having had a large number of pregnancies was associated with higher risk of CIN2+, with an almost three-fold increase for women with more than four pregnancies compared with those with 0–1 pregnancies. Several studies have reported the association of high parity with the risk of CIN2+, mainly in younger women [10,11,37–39], probably because of the prolonged period of exposure of the transformation zone to hrHPV and/or other cofactors in highly parous women [38]. The increased levels of estrogen and progesterone during pregnancy are probably responsible for the alterations in the transformation zone, potentially inducing a reduced immune response to HPV infection and influencing risk of persistence or progression [39]. A large collaborative epidemiological study on CC and hormonal contraceptives concluded that the risk of CC in current hormonal contraceptive users increases with the increasing duration of use [40]. Another study has suggested that neither high parity nor long-term use of hormonal contraceptives are associated with HPV prevalence, but may be involved in the transition from HPV infection to neoplastic cervical lesions [25].

Two large multicentric studies coordinated by the IARC have demonstrated that the education level is inversely and consistently associated with CC risk, but not with HPV prevalence [41]. It has also been found that the association between low socioeconomic status and level of education (especially no schooling) increases the risk for precancerous cervical lesions and invasive cancer in women with hrHPV [37]. In the present study, the socioesconomic status was not analyzed and the education level was not associated with CIN2+.

Regarding the knowledge, attitudes and practice related to HPV and CC, in a study conducted among women aged 30 years or more of a marginal riverside neighborhood of Asunción, Paraguay, only 10% of the women answered that they knew about HPV and 27% responded that Pap testing was associated with CC prevention [42]. Education, information and orientation about HPV infection, risk factors and also on the methods for the prevention of CC are necessary, to improve the strategy of prevention and control of CC. Furthermore, based on the association of the sexual behavior of the male partner observed in this study, we consider it appropriate to promote the participation of husbands or male partners of women in the information, orientation and education programs about prevention of HPV infection and the risk of hrHPVassociated genital cancer.

One of the strengths of our study was that the analysis of risk factors for CIN2+ was restricted to hrHPV+ women, a fact that allowed eliminating potential confounding by factors associated with the acquisition of infection. Another strength is that a standard protocols for data and specimen collection, laboratory testing and quality control, as well as a clinical management of HPV positive women that was validated by an international group of experts. Future analyses will provide additional detail, including HPV typing, multiple HPV infections and other sexually transmitted co-infecions, socio-economic factors, environmental and cultural factors, access to health centers and others.

In conclusion, this study provides the first population-based data on the risk factors for hrHPV infection and preneoplastic cervical lesions in an unvaccinated and not previously screened with HPV testing Paraguayan population. The sexual behavior of male partners was found to be associated with hrHPV infection, whereas underscreening practice and high number of pregnancies were found to be associated with CIN2+. Greater efforts to promote CC screening in our country are needed, particularly including educational campaigns on HPV infection and CC prevention targeting not only women but the whole population.

## Supporting information

S1 File Personal data form in english. (PDF)

S2 File Personal data form in spanish. (PDF)

S3 File Socio-demographic form in english. (PDF)

S4 File Socio-demographic form in spanish. (PDF)

S5 File Risk factors questionnaire form in english. (PDF)

S6 File Risk factors questionnaire form in spanish. (PDF)
