# Supplementary material for: Factors associated with high-risk human papillomavirus infection and high-grade cervical neoplasia: A population-based study in Paraguay
Source: PLoS One. 2019 Jun 27;14(6):e0218016. doi: 10.1371/journal.pone.0218016 (PMC6597051; doi:10.1371/journal.pone.0218016)
Supplement: S1 File — (PDF) [file pone.0218016.s001.pdf]

Participant Identification Barcode

PERSONAL DATA FORM
ESTAMPA

PD

1. **Clinic:** |_|_|	2. **Date completed**: |_|_|/|_|_|/|_|_|_|_| Day Month Year
3. **Name**	|_|_|_|_|_|_|_|_|_|_|_|_|_|_|_| **First Name** |_|_|_|_|_|_|_|_|_|_|_|_|_|_| **Second Name**<br>|_|_|_|_|_|_|_|_|_|_|_|_|_|_|_|_|_|_| **First Last Name**<br>|_|_|_|_|_|_|_|_|_|_|_|_|_|_|_|_|_|_| **Second Last Name**
4. **Date of Birth**	|_|_|/|_|_|/ |_|_|_|_| Day Month Year
5. **Identification**	|_|_|_|_|_|_|_|_|_|_|_|_|_|_|_|_|_|_|_|_|_| ________________
6. **Address**	____<br>____
7. **Exact geographic address**	**a. Province/Department:** ____________________<br>**b. Canton/Region:** ____________________<br>**c. District/ Town:** ____________________<br>**d. Locality:** ____________________
8. **Telephone numbers**	**a.** |_|_|_|_|_|_|_|_|_|_|_| **b.** |_|_|_|_|_|_|_|_|_|_|_| **c.** |_|_|_|_|_|_|_|_|_|_|_|
9. **E-mail address**	____
10. **Contact person who does not live in the same house**	|_|_|_|_|_|_|_|_|_|_|_|_|_|_| **First Name** |_|_|_|_|_|_|_|_|_|_|_|_|_| **Second Name**<br>|_|_|_|_|_|_|_|_|_|_|_|_|_|_|_|_| **First Last Name**<br>|_|_|_|_|_|_|_|_|_|_|_|_|_|_|_|_|_|_| **Second Last Name**<br>**Relation:** ____________________<br>**Telephone: 1.** |_|_|_|_|_|_|_|_|_|_|_|_|_| **2.** |_|_|_|_|_|_|_|_|_|_|_|_|_| **Address:** ____________________________________<br>____
11. **Code of the person who completes the form** |_|_|_|_|__________________	
