# Supplementary material for: Factors associated with high-risk human papillomavirus infection and high-grade cervical neoplasia: A population-based study in Paraguay
Source: PLoS One. 2019 Jun 27;14(6):e0218016. doi: 10.1371/journal.pone.0218016 (PMC6597051; doi:10.1371/journal.pone.0218016)
Supplement: S2 File — (PDF) [file pone.0218016.s002.pdf]

Participant Identification Barcode

SOCIO-DEMOGRAPHIC DATA
ESTAMPA

SDD

**Date of visit**: |__|__|/|__|__|/|__|__|__|__| Day Month Year	**Clinic:** |__|__|

I would like to take a few minutes to ask you some questions about your education and work

1.	**Can you read?**	1 Yes 2 No 9 NR	☐
2.	**Can you write?**	1 Yes 2 No 9 NR	☐
3.	**Did you ever attend school?**	1 Yes 2 No (go to 5) 9 NR (go to 5)	☐

4. **What is the highest grade or level of schooling you completed?** ☐

1	incomplete primary	5	incomplete undergraduate/university
2	complete primary	6	complete undergraduate/university
3	incomplete secondary	7	vocational/technical
4	complete secondary	9	NR/NK

5. **Which of the following descriptions express better the current situation of the head of household?(head of the household is the one who brings more money to the House)** ☐

1	works	5	retired
2	unemployed and looking for job	6	doing home tasks, taking care of the kids or other people
3	studying	7	other
4	permanently sick/handicapped	8	Neither working, nor looking for job
		9	NR/NK

6. **What is the occupation of the head of the household?** ☐☐

1	employee	8	temporary jobs
2	employed in domestic services	9	field work: herd ship, harvest, etc…
3	working in a program of social employment	10	worker without salary: e.g., works in a family company without salary
4	employer (with 5 or less employees)	11	other
5	employer (with more than 5 employees)	12	do not respond
6	professional or technician working independently	13	retired
7	working independent but not professional (at home, merchant, construction worker without formal contract of employment)	14	not applicable

7. **The water in your house:** ☐

1	is piped to the house	3	is out of the land
2	is within the land but out of the house	9	do not respond

8. **Do you have a bathroom?** ☐

1	Yes	9	Do not respond
2	No (skip to 10)		

9. **The bathroom has:**<br><br>1 button or chain toilet and water entraining 3 latrine (without water entraining)<br>2 toilet without button or chain but with water entraining (you use a bucket to flush water) 9 do not respond	☐
10. **In your house you have (mark all applicable)**<br><br>☐ car ☐ desktop computer<br>☐ microwave ☐ laptop computer<br>☐ cable TV ☐ washing machine<br>☐ none of the above	
11. **Finally, I would like to ask you if you have ever had a pap smear done?** — 1 Yes 2 No 9 NR/DK	☐
12. **When was your last pap smear done?**<br><br>1 less than 2 years ago 3 >5 years ago<br>2 2-5 years ago	☐
13. **Interviewer code: |__|__|__|__|____________________**	
