# Supplementary material for: Factors associated with high-risk human papillomavirus infection and high-grade cervical neoplasia: A population-based study in Paraguay
Source: PLoS One. 2019 Jun 27;14(6):e0218016. doi: 10.1371/journal.pone.0218016 (PMC6597051; doi:10.1371/journal.pone.0218016)
Supplement: S3 File — (PDF) [file pone.0218016.s003.pdf]

Código de barras participante

FORMULARIO DE DATOS PERSONALES
ESTAMPA

DP

1. **Clínica:** |__|__|	2. **Fecha completado**: |__|__|/|__|__|/|__|__|__|__| Día Mes Año
3. **Nombre**	|__|__|__|__|__|__|__|__|__|__|__|__|__|__|__| |__|__|__|__|__|__|__|__|__|__|__|__|__|__| **Primer Nombre** **Segundo Nombre**<br>|__|__|__|__|__|__|__|__|__|__|__|__|__|__|__|__|__|__| **Primer Apellido**<br>|__|__|__|__|__|__|__|__|__|__|__|__|__|__|__|__|__|__| **Segundo Apellido**
4. **Fecha de nacimiento**	|__|__|/|__|__|/ |__|__|__|__| Día Mes Año
5. **Identificación**	|__|__|__|__|__|__|__|__|__|__|__|__|__|__|__|__|__|__|__|__|__| ______________
6. **Dirección**	____<br>____
7. **Dirección Geográfica Exacta**	**a. Provincia/Departamento:** ____________________|__|__| **b. Cantón/Región:** ____________________|__|__| **c. Distrito/ Pueblo:** ____________________|__|__| **d. Localidad:** ____________________|__|__|
8. **Números telefónicos**	**a.** |__|__|__|__|__|__|__|__|__|__|__| **b.** |__|__|__|__|__|__|__|__|__|__|__| **c.** |__|__|__|__|__|__|__|__|__|__|__|
9. **Correo electrónico**	____
10. **Contacto personal que no viva en la misma casa**	|__|__|__|__|__|__|__|__|__|__|__|__|__| |__|__|__|__|__|__|__|__|__|__|__|__|__| **Primer Nombre** **Segundo Nombre**<br>|__|__|__|__|__|__|__|__|__|__|__|__|__|__|__|__| **Primer Apellido**<br>|__|__|__|__|__|__|__|__|__|__|__|__|__|__|__|__|__|__| **Segundo Apellido**<br>**Relación:** ____________________ |__|__| **Teléfono:** **1.** |__|__|__|__|__|__|__|__|__|__|__|__|__| **2.** |__|__|__|__|__|__|__|__|__|__|__|__|__| **Dirección:** ____________________________<br>____
11. **Código de la persona que completó este formulario** |__|__|__|__|__|____________________	
