# Supplementary material for: Factors associated with high-risk human papillomavirus infection and high-grade cervical neoplasia: A population-based study in Paraguay
Source: PLoS One. 2019 Jun 27;14(6):e0218016. doi: 10.1371/journal.pone.0218016 (PMC6597051; doi:10.1371/journal.pone.0218016)
Supplement: S4 File — (PDF) [file pone.0218016.s004.pdf]

# DATOS SOCIO DEMOGRÁFICOS
# ESTAMPA

Código de barras participante

| | |
|---|---|
| **Fecha de visita**: \|__\|__\|/\|__\|__\|/\|__\|__\|__\|__\|<br>Día Mes Año | **Clínica:** \|__\|__\| |

**Deseo tomar algunos minutos para hacerle algunas preguntas acerca de su educación y trabajo**

| | | | |
|---|---|---|---|
| 1. | **¿Sabe leer?** | 1 Sí 2 No 9 NR | ☐ |
| 2. | **¿Sabe escribir?** | 1 Sí 2 No 9 NR | ☐ |
| 3. | **¿Ha asistido alguna vez a la escuela?** | 1 Sí 2 No (pase a 5) 9 NR (pase a 5) | ☐ |

4. **¿Cuál fue el grado o nivel de escolaridad más alto que completó?** ☐

1 Primaria incompleta
2 Primaria completa
3 Secundaria incompleta
4 Secundaria completa
5 Pregrado/universidad incompleta
6 Pregrado/universidad completa
7 Vocacional/técnica
9 NR/NS

5. **¿Cuáles de las siguientes descripciones expresa mejor la situación actual del jefe de hogar (jefe de hogar es el que lleva más dinero a la casa)?** ☐

1 Trabaja
2 Desempleado buscando trabajo
3 Estudia
4 Enfermo permanente/discapacitado
5 Retirado
6 Hace tareas del hogar, cuida a los niños o a otras personas
7 Otro
8 Ni trabaja ni busca empleo
9 NS/NR

6. **¿Cuál es la ocupación del jefe de hogar?** ☐☐

1 Empleado
2 Empleado en oficios domésticos
3 Trabaja en un programa de empleo social
4 Empleador (con 5 empleados o menos)
5 Empleador (con más de 5 empleados)
6 Profesional o técnico trabajando Independiente
7 Trabajador independiente pero no profesional (en la casa, comerciante, trabajador de la construcción sin contrato formal de trabajo)
8 Trabajo temporal
9 Trabajo de campo: pastoreo, agricultura, etc…
10 Trabajador sin salario: trabaja en empresa familiar sin salario
11 Otro
12 No responde
13 Jubilado/a
14 No aplica

7. **El agua en su casa:** ☐

1 Llega por tubería a la casa
2 Está dentro del terreno pero fuera de la casa
3 Está fuera del terreno
9 No responde

8. **¿Tiene baño?** ☐

1 Sí
2 No (pase a 10)
9 No responde

| | | |
|---|---|---|
| 9. **El baño tiene:**<br>1 Sanitario con botón o cadena y arrastre de agua<br>2 Sanitario sin botón ni cadena pero con arrastre de agua (usa un balde para hacer correr el agua) | 3 Letrina (sin arrastre de agua)<br>9 No responde | ☐ |
| 10. **En su casa usted tiene: (marque todas las que apliquen)**<br>☐ Automóvil<br>☐ Horno microondas<br>☐ Televisión por cable | ☐ Computador de escritorio<br>☐ Computador portátil<br>☐ Lavadora<br>☐ Moto<br>☐ Ninguna de las anteriores | |
| 11. **Finalmente, quiero preguntarle si usted alguna vez se ha hecho un Papanicolaou o citología?** | 1 Sí 2 No 9 NR | ☐ |
| 12. **¿Cuándo se realizó su último Papanicolaou o citología?**<br>1 Hace menos de 2 años<br>2 Entre 2 a 5 años atrás | 3 Hace más de 5 años | ☐ |
| 13. **Código entrevistador: \|__\|__\|_______________________** | | |
