# Supplementary material for: Factors associated with high-risk human papillomavirus infection and high-grade cervical neoplasia: A population-based study in Paraguay
Source: PLoS One. 2019 Jun 27;14(6):e0218016. doi: 10.1371/journal.pone.0218016 (PMC6597051; doi:10.1371/journal.pone.0218016)
Supplement: S5 File — (PDF) [file pone.0218016.s005.pdf]

RISK FACTORS QUESTIONNAIRE ESTAMPA

Participant Identification Barcode

RFF

1. **Date of visit**: |__|__|/|__|__|/|__|__|__|__| (Day Month Year) **Clinic:** |__|__| **Code of interviewer:** |_|_|_|_|_______________

I want to take a few minutes to ask you some questions about yourself, your health and habits. This information could help us to better understand the relation between HPV infection and cervical lesions. I want to remind you that all information you provide is confidential and will be used only for the study. Finally, I want to remind you that you can refuse to respond any question without affecting your participation in the study.

2. I would like to ask you about your cigarette consumption. In your lifetime, have you ever smoked 100 cigarettes (5 boxes) or more? ☐

1 Yes, currently smoking
2 Yes, but do not smoke anymore
3 No, never [Go to question 7]
9 NR/NK [Go to question 7]

3. How old were you when you started smoking? (99 if NR/NK) |__|__|

4. On average, how many cigarettes did you or do you smoke? |_|_| per ☐

1 Day
2 Week
3 Month
4 Year
9 NR/NK
(If currently smoking, go to Question 7)

5. How old were you when you stopped smoking? (99 if NR/NK) |__|__|

6. For how many years did you smoke cigarettes? (Do not include time spans when she quit) (99 if NR/NK) |__|__| Years |__|__| Months

The next questions are about your sexual history. I realize that this is a sensitive subject, but it is very important for the study. Please take the time to recall this information as accurately as possible. I would like to remind you that the information you give will be confidential.

7. How old were you when you had your first menstruation? (99 if NR/NK) |__|__|

8. How old were you when you first had sexual intercourse with a man? (0 if never have had sexual intercourses [END])|__|__|

9. Throughout your life, with how many different men have you had sexual intercourse? |__|__|__|

If one man, go to question 13
More than one, go to question 11
NR/NK, go to question 10

10. Would you say they were: ☐

1 2 or 3
2 4 or 5
3 6 or 7
4 between 8 and 10
5 more than 10
9 NR/NK

11. During the past 12 months, with how many different men have you had sexual intercourse? |__|__|__|

If an exact number of men, go to question 16
If NR/NK, go to question 12

RISK FACTORS QUESTIONNAIRE
ESTAMPA

Participant Identification Barcode

RFF

**12. Would you say they were:**<br>1 2 or 3<br>2 4 or 5<br>3 6 or 7<br>4 between 8 and 10<br>5 more than 10<br>9 NR/NK<br>**Go to question 16**		☐
**13. As far as you know, what is the total number of women with whom your partner has had sexual intercourse, excluding you?**<br>If exact number of women, go to question 15<br>If Other, go to question 14		|__|__|__|
**14. Would you say they were:**<br>1 2 or 3<br>2 4 or 5<br>3 6 or 7<br>4 between 8 and 10<br>5 more than 10<br>9 NS/NR		☐
**15. During the last 12 months, have you had sexual intercourse?**	1 Yes 2 No 9 NR/NK	☐
**Finally, I want to ask you some questions about your pregnancies and the use of contraceptive methods, please try to remember this information with the major accuracy.**		
**16. Have you ever been pregnant?**	1 Yes 2 No 9 NR/NK<br>If No or NR/NK, go to question 18	☐
**17. How many times?**	|__|__|	
**18. Have you and your partner ever used a condom?**	1 Yes 2 No 9 NR/NK<br>If No or NR/NK, go to question 20	☐
**19. During the periods when you and your partner used a condom, did you use it**<br>1 Everytime you had sexual intercourse<br>2 Most of the time when you had sexual intercourse<br>3 Sometimes<br>4 Rarely<br>9 NR/NK		☐

RISK FACTORS QUESTIONNAIRE
ESTAMPA

Participant Identification Barcode

RFF

20. For each of the contraceptive methods listed below, please indicate to me if you have used it or not and if yes, please tell me how old you were when you started and stopped using it and for how long did you use it.

	No	Yes	Age at start	Age at stop	Time of use Months/Years	NR/NK
a. Pills	☐	☐	|__|__|	|__|__|	|__|__|/|__|__|	☐
b. Injections	☐	☐	|__|__|	|__|__|	|__|__|/|__|__|	☐
c. Implant s	☐	☐	|__|__|	|__|__|	|__|__|/|__|__|	☐
d. IUD	☐	☐	|__|__|	|__|__|	|__|__|/|__|__|	☐
e. Other, specify ________________________________			|__|__|	|__|__|	|__|__|/|__|__|	☐

21. Observations: ____
