# Supplementary material for: Factors associated with high-risk human papillomavirus infection and high-grade cervical neoplasia: A population-based study in Paraguay
Source: PLoS One. 2019 Jun 27;14(6):e0218016. doi: 10.1371/journal.pone.0218016 (PMC6597051; doi:10.1371/journal.pone.0218016)
Supplement: S6 File — (PDF) [file pone.0218016.s006.pdf]

CUESTIONARIO DE FACTORES DE RIESGO ESTAMPA

Código barras
Identificación
Participante

1. **Fecha visita**: |__|__|/|__|__|/|__|__|__|__| (Día Mes Año) **Clínica: |__|__|** **Código entrevistador:** |__|__|__|__|______________________

Quiero tomar unos minutos para hacerle algunas preguntas sobre usted, su salud y sus hábitos. Esta información podría ayudar a comprender mejor la relación entre la infección por VPH y las lesiones cervicales. Quiero recordarle que toda la información que usted proporcione es confidencial y será utilizada únicamente para el estudio. Por último quiero recordarle otra vez que puede negarse a responder cualquier pregunta, sin afectar su participación en el estudio.

2. Quisiera preguntarle acerca de su consumo de cigarrillos. ¿Durante su vida ha fumado al menos 100 cigarrillos (5 cajetillas) o más? ☐

1 Sí, fuma actualmente
2 Sí, pero ya no fuma
3 No, nunca [Vaya a pregunta 7]
9 NS/NR [Vaya a pregunta 7]

3. ¿A qué edad comenzó a fumar por primera vez? (99 si NS/NR) |___|___|

4. En promedio ¿cuántos cigarrillos fumaba o fuma? |__|__| por ☐

1 Día
2 Semana
3 Mes
4 Año
9 NS/NR
(Si fuma actualmente vaya a pregunta 7)

5. ¿A qué edad dejó de fumar? (99 si NS/NR) |___|___|

6. ¿Durante cuántos años fumó cigarrillos? (No Incluya lapsos de tiempo en que dejó de fumar) (99 si NS/NR) |___|___| años |___|___| meses

Las siguientes preguntas son acerca de su historial sexual. Sé que éste es un tema sensible, pero es importante para el estudio. Por favor, tómese el tiempo para recordar esta información con la mayor precisión posible. Me gustaría recordarle que la información que proporcione no se le dirá a nadie.

7. ¿A qué edad tuvo su primer periodo menstrual? (99 si NS/NR) |___|___|

8. ¿Qué edad tenía cuando tuvo su primera relación sexual con un hombre? (0 si nunca ha tenido relaciones sexuales [FIN])|___|___|

9. Durante su vida ¿con cuántos hombres diferentes ha tenido relaciones sexuales?

Si 1 hombre pase a pregunta número 13
Más de uno pase a pregunta 11 |___|___|___|
NS/NR vaya a pregunta 10

10. Diría que han sido ☐

1. 2 o 3
2. 4 o 5
3. 6 o 7
4. entre 8 y 10
5. Más de 10
9. NS/NR

11. Durante los últimos 12 meses ¿con cuántos hombres diferentes ha tenido relaciones sexuales?
Si un número exacto pase a la pregunta 16
Si NS/NR vaya a pregunta 12 |___|___|___|

CUESTIONARIO DE FACTORES DE RIESGO ESTAMPA

Código barras
Identificación
Participante

12. Diría que han sido

1. 2 o 3
2. 4 o 5
3. 6 o 7
4. entre 8 y 10
5. Más de 10
9. NS/NR

Pase a pregunta 16

☐

13. Que usted sepa ¿cuál es el número total de mujeres con las que su pareja ha tenido relaciones sexuales, sin incluirse a usted?

Si número exacto de mujeres pase a la pregunta 15

Si Otro, vaya a pregunta 14 |___|___|___|

14. Diría que han sido

1. 2 o 3
2. 4 o 5
3. 6 o 7
4. entre 8 y 10
5. Más de 10
9. NS/NR

☐

**15. Durante los últimos 12 meses ¿ha tenido relaciones sexuales?**	1 Sí 2 No 9 NS/NR	☐

Por último, quiero hacerle algunas preguntas sobre sus embarazos y el uso de métodos anticonceptivos, por favor, trate de recordar esta información con la mayor precisión posible.

**16. ¿Alguna vez ha estado embarazada?**	1 Sí 2 No 9 NS/NR<br>Si No o NS/NR Pase a pregunta 18	☐
**17. ¿Cuántas veces?**	|___|___|	
**18. ¿Usted y su última pareja, han usado alguna vez un condón?**	1 Sí 2 No 9 NS/NR<br>Si No o NS/NR Pase a pregunta 20	☐

19. Durante los períodos en que usted y su última pareja usaron condón, lo usaron

1 Cada vez que tuvieron relaciones sexuales
2 La mayoría de las veces que tuvieron relaciones sexuales
3 A veces
4 Rara vez
9 NS/NR

☐

CUESTIONARIO DE FACTORES DE RIESGO ESTAMPA

Código barras
Identificación
Participante

20. Para cada uno de los métodos anticonceptivos que se enumeran abajo, por favor indíqueme si usted lo ha usado o no, y en caso afirmativo, por favor dígame ¿qué edad tenía cuando lo empezó y lo dejó de usar y por cuánto tiempo lo usó?

	No	Si	Edad de inicio	Edad en que paró	Tiempo de uso Meses/Años	NR/NS
a. Pastillas	☐	☐	|__|__|	|__|__|	|__|__|/|__|__|	☐
b. Inyecciones	☐	☐	|__|__|	|__|__|	|__|__|/|__|__|	☐
c. Implantes	☐	☐	|__|__|	|__|__|	|__|__|/|__|__|	☐
d. DIU	☐	☐	|__|__|	|__|__|	|__|__|/|__|__|	☐
e. Otro, especifique ____________________			|__|__|	|__|__|	|__|__|/|__|__|	☐

21. ¿Ha padecido alguna infección de transmisión sexual? 1 Sí 2 No (Pase a pregunta 23) 9 NS/NR ☐

22. ¿Qué tipo de infección ha padecido? ☐

1 Sífilis
2 Herpes
3 VPH
4 Hepatitis B
5 Otros . especifique ____________________________

23. ¿Se higieniza luego del acto sexual? ☐

1 Siempre
2 Casi siempre
3 A veces
4 Rara vez
9 NS/NR

24. ¿Con qué se higieniza? ☐

1 Agua
2 Agua y jabón
3 Desinfectantes
4 Infusión de hierbas. Especificar tipo
5 Otros, especificar
9 NS/NR

24. a. Especificaciones: ____

25. Observaciones: ____
